# Influences of mental accounting on consumption decisions: asymmetric effect of a scarcity mindset

**DOI:** 10.3389/fpsyg.2023.1162916

**Published:** 2023-07-07

**Authors:** Lin Cheng, Yinqiang Yu, Yizhi Wang, Lei Zheng

**Affiliations:** ^1^School of Tourism, Huangshan University, Huangshan, China; ^2^School of Business, Macau University of Science and Technology, Taipa, Macao SAR, China; ^3^School of Economics and Management and School of Humanities and Social Sciences, Fuzhou University, Fuzhou, China; ^4^The Institute of Public Administration and Human Resources, Development Research Center of the State Council, Beijing, China

**Keywords:** scarcity mindset, mental accounting, hedonic consumption, utilitarian consumption, consumer behavior

## Abstract

A scarcity mindset is considered to impact consumer behaviors. Our research aimed to examine the moderating effect of the scarcity mindset on the relationship between mental accounting and hedonic (vs. utilitarian) consumption. We conducted an online experimental design (mental accounting: windfall gains vs. hard-earning gains; consumption: hedonic products vs. utilitarian products) and verified our hypotheses in two distinct samples: a student sample and an adult sample. Our results showed that consumers who received windfall gains tended to use it for hedonic consumption rather than utilitarian consumption. Intriguingly, such an effect was insignificant under a high level of a scarcity mindset but significant under a low level of the scarcity mindset. Moreover, consumers who received hard-earning gains tended to spend the money on utilitarian (vs. hedonic) consumption. However, we did not detect the impact of the scarcity mindset on such effects. Our research suggested an asymmetric effect of the scarcity mindset on hedonic (vs. utilitarian) consumption under two different mental accounts. It highlights the important role of the scarcity mindset in consumer behaviors, which leaves avenues for future research to understand marketing promotion strategies for distinct products.

## 1. Introduction

Consumers tend to place their money in different mental accounts, which further influence their consumption decisions (Thaler, 1985, 1999, 2008). Previous studies examined the influence of mental accounting on various consumer behaviors, such as hedonic consumption and utilitarian consumption (Babin et al., 1994; Alba and Williams, 2013). In fact, hedonic and utilitarian products are often presented simultaneously in previous studies (Hirschman and Holbrook, 1982; Babin et al., 1994) because the two products are often regarded to meet consumers' basic needs (Babin et al., 1994). However, recent studies showed that mental accounting seems to influence consumer behaviors that vary over populations and contexts (Cheema and Soman, 2006; Gou et al., 2013). One possible factor that may be related to mental accounting is a scarcity mindset because it has been considered to operate their mental budget to purchase scarce products (Hamilton et al., 2018). Particularly, the scarcity mindset may impact consumers' information processing toward their money, thus altering mental operations and their consequences on consumption behaviors. It would be possible that the influence of mental accounting on hedonic consumption vs. utilitarian consumption depends on the condition of a scarcity mindset. Based on the reasoning, two hypotheses were proposed. First, consumers are more likely to prefer hedonic (vs. utilitarian) products under the condition of windfall (vs. hard-earning) gains. Second, consumers with a high scarcity mindset are less likely than others to prefer hedonic products under windfalls condition. In order to verify our hypotheses, in this research, two samples were collected to further understand the effects of a scarcity mindset on hedonic (vs. utilitarian) consumption under different mental accounts.

### 1.1. Mental accounting and hedonic consumptions

Mental accounting defines a set of cognitive operations by consumers to code, categorize, and evaluate their financial activities (Thaler, 1999). This behavioral economic concept explains how consumers treat personal money and make purchase decisions, depending on the way they earned the money, how they intended to use the money, and their subjective value of the money (Thaler, 1985, 2008). For example, consumers tend to segregate gains by placing them into different accounts with distinct functions and uses (Thaler, 2008; Zhang and Sussman, 2018). Notably, the money assigned in the distinct mental account is considered to be different and irreplaceable. Such a nonfungibility effect is considered the fundamental feather of mental accounting (Thaler, 1985; Sui et al., 2021). Although the operation rules of mental accounting often violate the classical economic principle of fungibility, consumers are susceptible to mental accounting when they make purchase decisions (Cheema and Soman, 2006; Xiao and O'Neill, 2018; Sui et al., 2021).

Since Thaler proposed the theory of mental accounting (Thaler, 1999), a wealth of evidence has shown that consumers make purchase decisions within the limited spending and saving categories in a budget system (Arkes et al., 2008; Reinholtz et al., 2015; Hossain et al., 2018; Sui et al., 2021). Notably, consumers tend to label money based on the context in which it was obtained (Thaler, 1985; Levav and McGraw, 2009). It creates “income mental accounts” that determine their consumption behaviors in a way that “matches” the budget rules (Levav and McGraw, 2009). For example, consumers often place windfalls in a distinct mental account, in which the money is often used to purchase items they would not buy regularly, such as hedonic products (Milkman and Beshears, 2009) and luxuries (Rajagopal and Rha, 2009). In fact, hedonic consumption is closely related to the affective and experiential aspects of purchases (Babin et al., 1994; Alba and Williams, 2013). Consumers engage in hedonic consumption to maximize pleasure and happiness because these products are perceived as relatively more enjoyable, fun, and fantasy (Hirschman and Holbrook, 1982). According to the mindsponge theory (Vuong and Napier, 2015; Vuong et al., 2022; Vuong, 2023), consumers may view windfall gains from an easy labor trade-off perspective, which may reduce the perceived cost of hedonic consumption. However, for hard-earned money, consumers usually carefully/rationally weigh desires for hedonic consumption with their expenditure of effort. In line with the mental accounting theory, when consumers obtain “happy money,” such as windfalls, they are more likely to spend on hedonic products.

However, the normal/hard earning is thought to assign to a generic mental account that is generally used for utilitarian consumption (Gou et al., 2013; Sui et al., 2021). Particularly, utilitarian products are often presented with hedonic products (Hirschman and Holbrook, 1982; Babin et al., 1994). It is often perceived as relatively more necessary, useful, and functional (Alba and Williams, 2013). Both consumptions are considered as primarily consumption activities that fulfill consumers' basic needs (Babin et al., 1994). In fact, consumers seem to make more effort in comparing the benefits of hedonic consumption compared to utilitarian consumption because the expected happiness and pleasure caused by hedonic products are more difficult to evaluate (Alba and Williams, 2013; Liu and Chou, 2020). Specifically, hedonic consumption can elicit a sense of guilt when such activity comes in association with wastefulness (Liu and Chou, 2017, 2020). In fact, consumers tend to avoid hedonic consumption when the money comes from a negative circumstance. For example, when consumers are bequeathed money from their dead relatives, the money is labeled as sadness and is usually used for pragmatic (i.e., utilitarian) rather than hedonic consumption (Levav and McGraw, 2009). Similarly, financial compensation due to their child's wrongful death suits is often labeled as “blood money,” which is more probably used for donation or charity (Zelizer, 1994). In fact, studies have shown that how consumers obtaining money affects the way it is used for consumption (Sui et al., 2021).

### 1.2. Scarcity mindset and consumer behaviors

A scarcity mindset is a belief that resources are limited and consumers can never have enough to meet their requirements (Mani et al., 2013; Meuris and Leana, 2015; Cannon et al., 2019; Goldsmith et al., 2021). Their mind is focused on scarce resource to the extent that they ignore anything else, no matter how hard they try (Shah et al., 2012; Mani et al., 2013; Roux et al., 2015; Li et al., 2021). Notably, according to these scarcity studies, promotion strategies and environmental factors, such as limited-time scarcity (Kristofferson et al., 2016), limited-quantity scarcity (Jang et al., 2015), and financial pressures (Mani et al., 2013; Probst et al., 2020), have also been found to be related to consumption behaviors (Hamilton et al., 2018; Goldsmith et al., 2021). This is partly because the scarcity mindset shifts attention to activities where scarcity is dominant and partly because it creates a cognitive load that prevents consumers from figuring out the optimal choices (Cannon et al., 2019; Huijsmans et al., 2019).

Despite several studies found that scarcity-induced consumers tend to be more engaged in games and exhibit more product use creativity (Shah et al., 2012; Mehta and Zhu, 2016), a scarcity mindset is considered to be negative and causes the low quality of life (Kristofferson et al., 2016; Hamilton et al., 2018; Goldsmith et al., 2021). This is in line with the mindsponge theory, which states that our mind perceives and processes resource scarcity information, which alters psychological states in response to environmental conditions (Vuong, 2023). In particular, the information is first collected and filtered by the mind and processed based on subjective cost–benefit judgment within the mind. It is considered to be influenced by the values, especially the core values in the mindset. Ultimately, the information is absorbed into the mind, thus resulting in behavioral responses. Accordingly, many studies have found that a scarcity mindset is related to irrational behaviors, such as impulsive purchases (Mani et al., 2013), overborrowing (Shah et al., 2015), and aggressive behaviors (Roux et al., 2015). Thus, consumers with the scarcity mindset are characterized by insufficient resources and a lack of control (Hamilton et al., 2018; Huijsmans et al., 2019).

Although it seems that consumers with a scarcity mindset tend to save money, a recent study suggested that consumers often save money and operate their limited budget for a major purchase (Hamilton et al., 2018). In fact, another study suggested that those who experienced scarcity-related childhood environments are more likely than others to spend money rather than save money to meet their current needs (Griskevicius et al., 2013). Shah et al. (2012) posit that consumers often place their savings in a specific account for expenses rather than a generic account. Since the scarcity mindset leads them to make financial decisions based on local convenience to fulfill their immediate needs (Shah et al., 2012; Kristofferson et al., 2016; Hamilton et al., 2018; Goldsmith et al., 2020, 2021), it may change the way consumers operate their financial resources in terms of mental accounting.

### 1.3. Scarcity mindset as the boundary condition

Although mental accounting theory is widely accepted today, consumers are considered to flexibly place money in different mental accounts under ambiguous conditions, which challenges the nonfungibility effect of this theory (Cheema and Soman, 2006; Gou et al., 2013). For example, food delivery could be assigned in either the utilitarian or hedonic consumption account because it is pragmatic as daily lunch and is hedonic as a wonderful meal, respectively. In fact, personality traits have also been thought to impact the way consumers assign money in mental accounts (Gou et al., 2013), such as conscientiousness, non-planning impulsivity, financial knowledge, and short-time orientation (Antonides et al., 2011; Muehlbacher and Kirchler, 2019; Olsen et al., 2019).

As a scarcity mindset is considered to affect the way consumers operate their resources (Shah et al., 2012), we suggest that the scarcity mindset may affect mental accounting. In particular, consumers with a high scarcity mindset tend to reduce hedonic consumption compared to others under the windfalls condition. This is because the scarcity mindset shifts consumers' attention to their immediate needs and makes efforts to engage in scarcity-related tasks (Shah et al., 2012; Kristofferson et al., 2016; Hamilton et al., 2018; Goldsmith et al., 2020, 2021). As utilitarian consumption is usually viewed as the means to achieve tangible goals (Alba and Williams, 2013; Liu and Chou, 2020), it is more likely to fulfill consumers' basic needs in terms of a scarcity mindset. However, in hedonic consumption, it emphasizes the emotional and experiential aspects of the consumption. Consumers seldom view momentary enjoyment as scarce resources, but more often perceive money and time as scarce and limited. For example, if a consumer has a hectic work schedule (i.e., time scarcity), it is less likely for him/her to prefer traveling to another country, even if under the condition of winning a lottery.

### 1.4. The present study

In this research, we conducted two experiments: one college student sample and one adult sample. We aimed to repeatedly test our hypotheses in both samples. Given the theory of mental accounting (Thaler, 1999), as we have mentioned above, consumers are thought to spend “happy money” (e.g., windfalls) for hedonic consumption but use “unhappy money” (e.g., hard-earning money) for utilitarian consumption. Thus, our first hypothesis is that consumers are more likely to prefer hedonic (vs. utilitarian) products under the condition of windfall (vs. hard earning) gains. Based on the scarcity theory, scarce information (objective world) can cause a scarcity mindset (subjective world) and further impact consumers' behavioral responses (Goldsmith et al., 2021). Thus, the scarcity mindset may affect the financial operations within the hedonic mental accounts rather than that in the utilitarian mental accounts. Based on the above reasoning, we hypothesized that the scarcity mindset moderates the effects of mental accounting on hedonic (vs. utilitarian) consumption. In particular, consumers with a high scarcity mindset are less likely than others to prefer hedonic products under windfalls condition.

## 2. Methods

### 2.1. Participants

Our research collected two samples to repeatedly verify our hypotheses: one is a college student sample (all age > 18 years) and another is an adult sample. We recruited 319 students (156 female students, mean age = 19.79 ± 1.26 years) from a university in Fujian, China. They participated in the online experiment for extra credit in a psychological course. They all finished the study at an online survey platform (www.wjx.cn). In the adult sample, 294 adults (131 women, mean age = 48.07 ± 6.58 years) were recruited from western China. They were recruited on a social media platform (WeChat) through a link shared by their relatives, which contain the recruitment requirements and a QR code. The code redirected them to the survey platform. Once they finished the experiment, they were acknowledged for their participation and also received two yuan RMB.

### 2.2. Measures

#### 2.2.1. Scarcity mindset

Three items were adapted from previous studies to measure the scarcity mindset (Pitesa and Thau, 2018). The statements were “I feel essential resources (money, food, and water) are scarce,” “I think shortages of essential resources are possible,” and “I am concerned about my long-term ability to acquire essential resources.” Participants were asked to rate on a 5-point Likert scale (1 = “strongly disagree” to 5 = “strongly agree”). The Cronbach's alpha was 0.88 for sample 1 and 0.83 for sample 2.

### 2.3. Design and procedure

Our research utilized a 2 (mental account: windfall gains vs. hard-earned money) × 2 (consumption type: hedonic vs. utilitarian consumption) between-subject design. The protocol was approved by the institutional review board of the corresponding author's university. All participants first finished the scarcity mindset scale after they read and gave their online consent. Next, they were randomly assigned to either the windfall gains or the hard-earned money group. In the student sample, they received the following instructions and were asked to make a decision for themselves:

*You usually receive living costs from your parents on the first day of each month. Today, you received 100 Yuan RMB because of [winning the lottery/ one-day part-time job]. Which do you prefer to spend the money on? Depositing the money to your canteen card or having dinner at a high-quality restaurant*.

For the adult sample, they received the following instructions, and were also asked to choose one option for themselves:

*You usually receive your salary on the last day of each month. Today, you received 3,000 Yuan RMB because of [winning the lottery/overtime work]. Which do you prefer to spend the money on? Buying a large home appliance (e.g., refrigerators) or traveling out of town (e.g., Booking a high-quality hotel)*.

After that, they were informed to complete their demographic information and were debriefed via an online page.

## 3. Results

**Figure 1:**
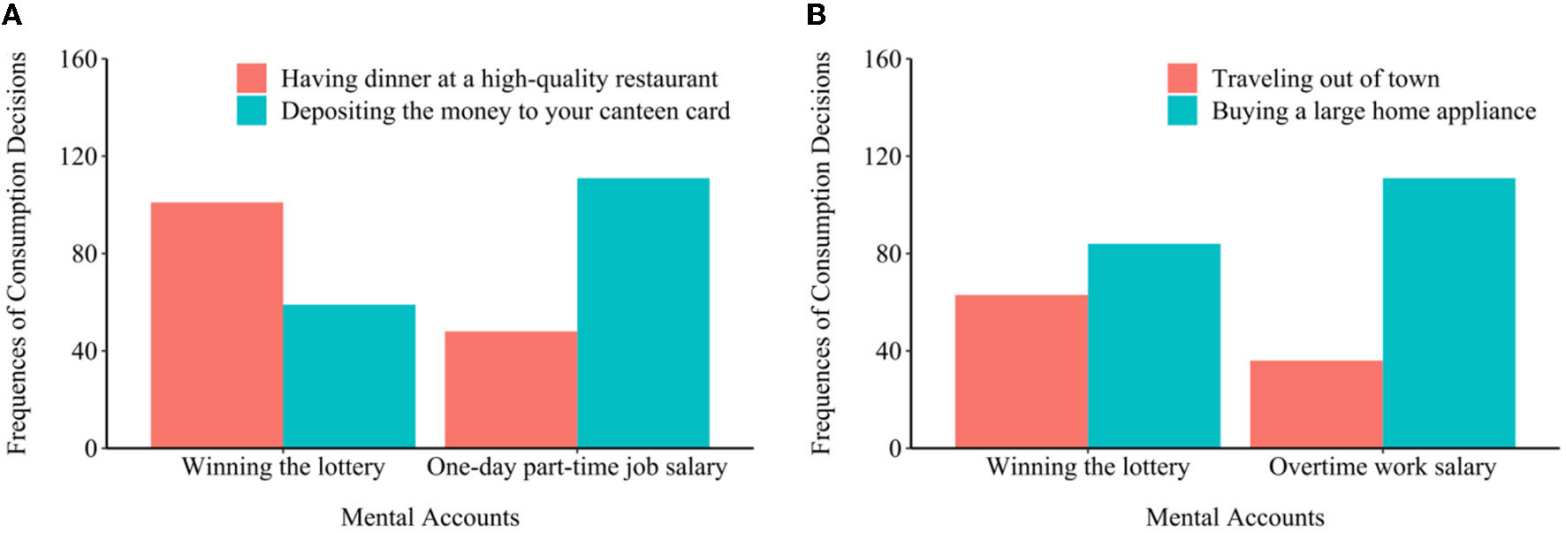
Influences of mental accounting on consumption decisions among college students **(A)** and adults **(B)**.

To test the effects of mental accounts on consumption decisions, chi-square tests were conducted for the two samples. As hypothesis 1, our results showed that consumers who received windfall gains tended to choose experience consumption, whereas others who received hard-earned money tended to prefer utilitarian consumption [student sample: χ^2^(1) = 33.45, Cohen's *d* = 0.68, *p* < 0.001; adult sample: χ^2^(1) = 10.30, Cohen's *d* = 0.38, *p* = 0.001]. As shown in Figure 1A, student consumers were more likely to have dinner at a high-quality restaurant if they received money by winning a lottery, but they were likely to deposit the money to their canteen card if it was their part-time salary. Similarly, for adults, they tended to spend their windfall money on buying experiences (booking a high-quality hotel) rather than assets (i.e., a large home appliance; Figure 1B).

Next, we conducted logistic regression models to examine the effects of a scarcity mindset on hedonic (vs. utilitarian) consumption. Our results showed that there were significant main effects of the scarcity mindset on consumption decisions for the student sample [*B* = −0.39, SE = 0.13, 95% CI (−0.64, −0.14), *p* = 0.003] but not for the adult sample [*B* = −0.16, SE = 0.13, 95% CI (−0.41, 0.09), *p* = 0.212].

**Figure 2:**
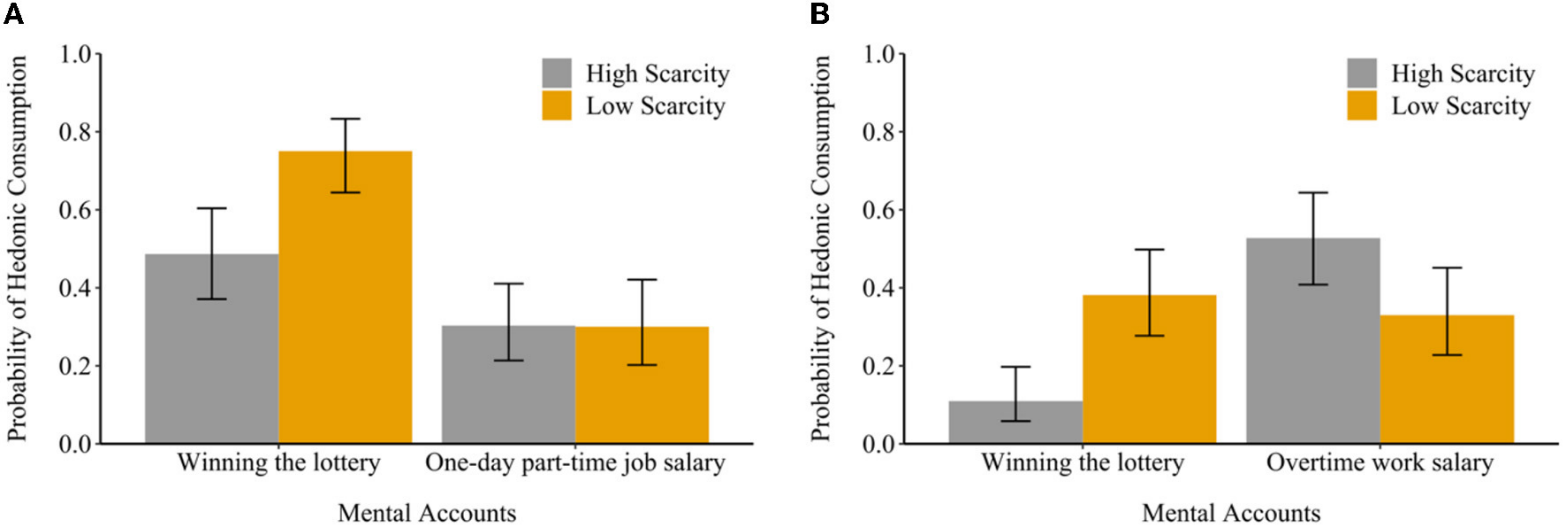
Moderating effects of a scarcity mindset on the influences of mental accounting on consumption decisions among college students **(A)** and adults **(B)**.

In addition, logistic interaction models were conducted to examine the moderating effects of a scarcity mindset on the influences of mental accounts on consumption decisions. In line with hypothesis 2, this research found that scarcity mindset altered the influences of windfall gains on hedonic (vs. utilitarian) consumption [student sample: *B* = −0.66, SE = 0.30, 95%CI (−1.25, −0.07), *p* = 0.026, Figure 2A; adult sample: *B* = −1.28, SE = 0.30, 95% CI (−1.87, −0.69), *p* < 0.001, Figure 2B]. In particular, consumers with a high scarcity mindset were less likely to spend windfall gains on hedonic consumption (i.e., having dinner in a high-quality restaurant or traveling out of town). However, according to hard-earned money, a scarcity mindset did not seem to influence their choices. It suggested that scarcity mindset-impacted consumption decisions (i.e., hedonic vs. utilitarian consumption) vary when the spending came from different mental accounts.

## 4. Discussion

Using two samples, our research found an asymmetric effect of a scarcity mindset on consumers' preference toward hedonic (vs. utilitarian) consumption within two different mental accounts. As shown in previous studies (Li et al., 2014; Sui et al., 2021), consumers tend to prefer hedonic consumption within windfall gains mental account. However, such effects seem to be inhibited among consumers with a high scarcity mindset. According to utilitarian consumption within hard-earning gains mental account, it seems that the scarcity mindset has no impact on such a generic account.

### 4.1. Findings

Our research repeated previous findings that consumers tend to use “happy money” for hedonic consumption but use “unhappy money” for utilitarian consumption. However, in the adult sample, consumers appear to prefer utilitarian consumption regardless of two mental accounts. One possible reason is that the amount of gains is larger than the monthly disposable income of 2020 (~2,682.42 yuan RMB) in China (National Bureau of Statistics of China, 2021). The windfall gains are larger than the average monthly disposable income, which may enhance the pain of payment for a momentary enjoyment. In fact, consumers usually place more weight on utilitarian consumption (Kahnx et al., 1997). This is especially true for Chinese consumers because Chinese culture values thrift and hard work rather than extravagance and indulgence (Okada, 2005; Liu and Chou, 2020). Particularly, a scarcity mindset often intensifies the sense of competition (Zheng et al., 2023) and leads consumers to focus on the potential value of the purchase (Kristofferson et al., 2016). Thus, the scarcity mindset may increase the sense of payment pain and inhibit the hedonic consumptions because adults usually have a higher sense of responsibility compared to students. In line with that, our results showed that the scarcity mindset reduced the hedonic consumption preferences of the adult sample. Thus, consumers are more likely to prefer utilitarian (vs. hedonic) consumption since hedonic consumption elicits stronger pain of paying and suppresses the expected happiness (Kahnx et al., 1997). Conversely, for the student sample, they receive a relatively smaller windfall gain, which comes with a weaker pain of payment. Nevertheless, future studies should consider the effects of gain's size on consumer decision makings within different mental accounts.

Another possible explanation is that students receive living costs from their parents, whereas these adults have to earn money to support their families. As their sources of living costs in a generic account are different, these attitudes toward windfall gains may be affected by such money (Xiao and O'Neill, 2018; Zhang and Sussman, 2018). It is possible that the two populations place the windfall gains in different mental accounts, which subsequently affects their subsequent consumption decisions. For example, students may place the windfall gains in the mental account regarding hedonic expenses, and parents may view the money as family income and assign it to their generic account that is used for repaying debts and daily expenses. In fact, adult consumers usually have a higher sense of responsibility compared to students. It leads them to consider more payment pain in hedonic consumption. Our results provide evidence to the mindsponge theory (Vuong and Napier, 2015; Vuong et al., 2022; Vuong, 2023), and their behavioral responses were moderated by their value, environment, and culture. As adults often have to support the growth of their offspring, these family roles may affect their attitudes toward the windfall gains, which further alters the consumption decision.

It is worth noting that our research found that the effects of a scarcity mindset on mental accountings are asymmetry. Our results showed that the scarcity mindset moderated the relationship between windfall gains and hedonic (vs. utilitarian) consumption. It suggests that consumers who perceive their resources are insufficient, tend to reduce hedonic consumption but increase utilitarian consumption. Our findings support the “malleable mental accounting” hypothesis that there is an individual difference according to financial operations within mental accounting (Cheema and Soman, 2006; Gou et al., 2013; Liu and Chou, 2020). The windfall gains may be viewed as different money and be assigned to distinct mental accounts due to their psychological perspective. For example, if a lottery gain comes from a just dead relative, consumers may view it as “pain money” and are less likely to use it for hedonic consumption in such a context (Zelizer, 1994; Levav and McGraw, 2009). Similarly, when consumers wonder how they will pay for their living expenses, a windfall gain is probably placed in a pragmatic mental account. Thus, our research showed that the scarcity mindset affects consumption decisions within the hedonic mental account but does not alter consumption decisions within the utilitarian mental account.

### 4.2. Implications

Many studies have shown that scarcity promotion affects consumer behaviors (Kristofferson et al., 2016; Hamilton et al., 2018; Goldsmith et al., 2020, 2021). This research highlights the important role of the mindsponge theory in explaining consumption decisions (Vuong et al., 2022; Vuong, 2023). Environmental information, such as scarcity promotion, may alter consumption decisions depending on product types. According to utilitarian consumption, a scarcity-induced strategy tends to promote competition orientation and aggression regardless of limited-time or limited-quantity promotion (Kristofferson et al., 2016) because utilitarian products can fulfill their psychological needs caused by scarcity promotion. In fact, scarcity promotion is thought to promote creativity in using utilitarian products because of the limited-quantity constraints (Mehta and Zhu, 2016). However, for hedonic consumption, scarcity promotion may lead to negative outcomes since our results suggest an inhibitory effect of a scarcity mindset on hedonic consumption. Based on the reasoning, differentiated promotion strategies should be distributed for utilitarian and hedonic consumption. Our research leaves avenues for future research on utilitarian vs. hedonic consumption within mental accounting, which can have important implications for companies to take appropriate marketing strategies for distinct products.

In addition, hedonic consumption is thought to promote wellbeing by improving consumers' life satisfaction (Zhong and Mitchell, 2010; Alba and Williams, 2013). In fact, behavioral responses are influenced by the values within the mind, especially the core values in the mindset (Vuong et al., 2022; Vuong, 2023). In line with that, previous studies highlight that low-cost hedonic products elicit a sense of satisfaction with relevant life domains (Zhong and Mitchell, 2010; Liu and Chou, 2020). Demand theory posits that people engage in consumption behaviors to maximize their happiness (Suranyi-Unger, 1981). However, our research showed that consumers with a scarcity mindset are more likely to prefer utilitarian consumption rather than hedonic consumption, which perhaps departs from the consumption goals. Therefore, with respect to governance, programs decreasing the scarcity mindset may help consumers shift their attention to experiential products rather than pragmatic products, such as inspiring consumers to enjoy happy moments instead of encouraging social comparison.

### 4.3. Limitations and future directions

There are several limitations to this research. First, the scarcity mindset was evaluated by using a self-reported measurement adapted from a previous research study (Pitesa and Thau, 2018). Although the reliability of the scarcity mindset scale was acceptable, a validity examination was needed. In fact, a scarcity mindset can be caused by many factors, such as time and money. However, our research merely provides evidence about the effects of a general scarcity mindset on consumption decisions. Thus, it is unclear which kind of resource constraint contributes more to consumers' preferences. As source funding may play a critical role in consumption decisions, we suggest further studies to conduct a field experiment to validate our findings. Second, this research recruited participants from two different regions in China. Although it may increase the generalization of our findings, there are many factors (e.g., socio-economic status) that may shape consumer behavior and mindset. Moreover, this study merely collected their age and gender, and further studies should include other demographic variables as covariables. As there is uneven development of regional economies, it may increase the scarcity mindset. We suggest future studies to test the results over regions and across countries. Third, although our research provides several possible explanations, psychological mechanisms underlying the effects of a scarcity mindset on mental accountings are still unknown. Future studies may consider other explanations to further tap into the relationship between the scarcity mindset and mental accounting.

## 5. Conclusion

Our research indicated an asymmetric effect of a scarcity mindset on hedonic consumption vs. utilitarian consumption under two different mental accounts. Consumers are more likely to prefer hedonic consumption when they receive windfall gains. Our results showed that such an effect is inhibited under a high level of a scarcity mindset but not under a low level of the scarcity mindset. However, according to hard-earning gains, we did not detect a significant difference in hedonic (vs. utilitarian) consumption under different levels of the scarcity mindset.

## Data availability statement

The raw data supporting the conclusions of this article will be made available by the authors, without undue reservation.

## Ethics statement

The studies involving human participants were reviewed and approved by Fuzhou University. The patients/participants provided their written informed consent to participate in this study.

## Author contributions

LC and LZ: conceptualization. YY: methodology and visualization. YY and YW: formal analysis. LZ: investigation, writing—review and editing, project administration, and funding acquisition. LC: writing—original draft preparation. LZ and YW: supervision. All authors have read and agreed to the published version of the manuscript.
